# The Path Planning and Location Method of Inspection Robot in a Large Storage Tank Bottom

**DOI:** 10.1155/2023/3029545

**Published:** 2023-03-02

**Authors:** Yinchu Wang, Haijiang Zhu, Yue Yu, Bin Hu

**Affiliations:** ^1^College of Information Science and Technology, Beijing University of Chemical Technology, Beijing 100029, China; ^2^China Special Equipment Inspection and Research Institute, Beijing 100026, China

## Abstract

With the development of robot technology, inspection robots have been applied to the defect detection of large tanks. However, the existing path planning algorithm of the tank bottom detection robot is easy to fall into the local minimum, and the path is not smooth. Besides, the positioning of the tank bottom detection robot is not accurate. This article proposes a path planning and location algorithm for the large tank bottom detection robot. Specifically, we design a preset spiral path according to the shape of the tank bottom, and a rotating potential field (RPF) near the obstacle is added to avoid the problem of path planning falling into a local minimum. We obtained accurate and smooth planning results. Compared with the state-of-the-art, the RPF method reduced the average RMSE by 9.49%. In addition, by measuring the acoustic emission distance, the three-point positioning algorithm can be used to achieve the calculation of the robot position detection in the proposed method, and the average positioning error on the spiral path is only 0.0748 ± 0.0032.

## 1. Introduction

Oil storage tank is an essential and important infrastructure in the petroleum, chemical, and other industries [1]. And the tank may be deformed or leaked under the action of various gas-liquid corrosion and stress changes [2]. If there are potential safety hazards in the oil storage tank, it may cause huge losses [3]. In the recent years, China oriental chemical plant “6.27,” Huangdao oil depot “8.12,” Dalian Petrochemical Company “8.29,” Shandong Shida Technology Company “7.16,” and other accidents indicate that China's oil storage tank accidents are still in a state of multiple occurrences [4]. Therefore, the detection and data collection of oil storage tank is a challenging task.

The metal oil tank is a vessel welded with a steel plate. High strength low alloy steel is used for large volume oil tanks with a capacity of more than 10,000 m^3^ [5]. The common shapes of metal oil tanks are vertical cylindrical, horizontal cylindrical, and spherical. For the most common vertical cylindrical oil tank, the tank bottom is horizontal and round, and a small part of the area is covered with obstacles such as pipes, valves, and oil sludge. Traditional tank bottom detection methods required professionals to carry special equipment into the tank body [6]. This detection method has large potential safety hazards and many processes. To mitigate the risk and simplify the process, Leon-Rodriguez described the design of an umbilical-free mobile nondestructive testing (NDT) climbing robot, and the robot was made transitions between the surfaces [7]. Zhang proposed a new climbing robot with a simplified motion mode and a strong load capacity [8]. The designed robot has high mobility and can successfully realize the climbing movement, but it cannot meet the demand of omnidirectional movement. After that, Li developed a novel Mecanum omnidirectional climbing robot for tanks inspection [9]. Nevertheless, the application of climbing robot is limited by the poor adsorption capacity. Zhang investigated an innovative wall-climbing robot system based on magnetic circuit optimization [10]. But manual assistance is required during detection. Subsequently, Feng explored a wall-climbing robot with the fusion welding forming model based on BP neural network for automatic welding of the island spherical tank [11]. However, the motion of the robot at the tank bottom has not been considered. To solve this problem, Chrysalidis introduced and analyzed various robot systems for cleaning residual oil at the tank bottom [12]. But these residual oil cleaning robot systems still need manual assistance. Therefore, Chang established a wheeled robot with a magnetic flux leakage testing device [13]. And yet, the circular motion suitable for the tank bottom shape is not considered. Thus, Mondal proposed an adjustable circular shape robot [14]. The rotational module is designed to allow the wheels to rotate and be able to go in a circular motion. Nevertheless, obstacle avoidance is not considered in this literature. In summary, how to realize automatic obstacle avoidance and dynamic path planning still needs further improvement.

With the development of intelligent automobile and robot, path planning has attracted more and more attention. Traditional path planning algorithms include A-Star, Dijkstra, D-Star Lite, RRT, neural network, intelligence algorithm, and artificial potential field method. A-Star is an effective direct search algorithm and is broadly applied. With the increase of nodes, the efficiency decreases significantly [15, 16]. Dijkstra solved the shortest path problem through constructing a directed graph and the optimal solution was obtained [17, 18]. However, the space occupied by Dijkstra is large. D-star Lite searches path nodes by maintaining one priority queue and has dynamic planning capability [19, 20]. The disadvantage is low efficiency when the state space is large. In RRT, random spanning trees and searching paths were generated. Although the algorithm principle is simple, the planned path cannot be guaranteed to be the optimal path [21, 22]. The solution was optimized in neural network through designing multiple neurons with nonlinear mapping capability and connecting them with weight coefficients. Here, nonlinear mapping and parallel processing were realized but the training time is too long [23, 24]. And path planning based on the intelligence algorithm may avoid the problem of local minimum and they are computationally expensive and difficult to solve the problem of high dimension [25]. In comparison, the artificial potential field method is a common method with high efficiency and a wide application range for robot path planning [26]. The path planned by the potential field method is generally smooth and safe, but this method has local minimum problem. To solve the problem of falling into the minimum, many scholars have done research. Sun presented a dynamic window approach and defined a danger index in the speed field for moving object avoidance. But the problem of inaccessibility of the target is not considered [27]. On this basis, Liu improved a potential field path planning method based on the genetic algorithm, where the genetic algorithm was used to optimize the combined potential field function of gravity and repulsion and found the lowest point of potential energy directly so as to determine the step size and moving direction of the robot [28]. However, the security of path planning results is still insufficient. Orozco-Rosas proposed a membrane evolutionary artificial potential filed (memEAPF) approach with combined membrane computing with a genetic algorithm and improve the security of planned paths [29]. Nevertheless, the oscillation between obstacles and concave obstacle problems is still not considered. Aiming at these problems, Lin constructed an artificial potential field path planning model based on decision tree through utilizing the advantages of decision tree in rule expression and extraction, in which this algorithm realized the real-time and accurate identification of current behavior and fast decision-making of next time behavior in path planning [30]. With the complexity of the improved methods, the computational complexity is further increased. Thus, Tian proposed a method to construct a guided potential field in the virtual guiding pipeline. The algorithm complexity is reduced and the ability to avoid local minima is improved [31]. However, the path planning results are not smooth. Orozco-Rosas proposed a QAPF learning algorithm combining Q-Learning and the artificial potential field to obtain smooth results [32]. In addition, the virtual target (VT) method is also proposed to make the path smoother [33]. Virtual targets are designed according to nearest obstacle and generate additional gravity in the VT method. Although the VT method effectively smooths the paths, many virtual targets need to be designed for spiral path planning, which affects the efficiency of path planning. Thus, Zhao proposed an improved artificial potential field (IAPF) method (state-of-the-art) with designed additional gravity according to the direction of the local minimum point [34]. Although the IAPF method can effectively jump out of the local minimum point, the cost is that there are redundant path points on some paths, which reduces the smoothness of the planning results and increases unnecessary movement. Therefore, on the basis of avoiding local minimum points, how to consider smoothness and efficiency of the path planning algorithm needs to be further studied.

Besides, the accuracy and effectiveness of the positioning algorithm are crucial to move the robot accurately according to the path planning results. Until now, the existing indoor location methods have limited positioning accuracy and different costs when applied in oil tanks, such as the methods based on Wi-Fi, WLAN, ZigBee, Bluetooth, and ultra-wideband (UWB). The wireless signal in Wi-Fi is vulnerable to interference and reflection, resulting in limited positioning accuracy [35]. Shadow and multipath effect exists in WLAN [36]. ZigBee is easy to cause multipath effect and abnormal signal attenuation due to the influence of the nearby environment [37]. Bluetooth has a small coverage and its accuracy is affected by beacon density [38]. UWB can ensure the positioning accuracy but the equipment cost is high [39]. As a result, a high-precision and cost-acceptable positioning method needs to be considered.

At present, the storage tank in China has been operating for a long time. Tank maintenance and health status monitoring is an urgent task. Due to the lack of the automatic detection equipment, the detection of tank bottom plate is labor-intensive. And the detection efficiency is very low. Furthermore, working for a long time in the sealed space of the storage tank is harmful to the health of the staff.

To raise the inspection efficiency of the large storage tank bottom, this article intends to propose the path planning and positioning algorithm of the large-scale tank bottom detection robot. The main contributions of this article are as follows:

- The local minimum and the lack of smooth planning results are two main problems in the traditional artificial potential field method. The proposed rotating potential field (RPF) method can effectively avoid the local minimum trap and obtain smooth planning results while avoiding obstacles. Combined with the preset spiral path, it can be used for automatic path planning of the tank bottom detection robot.
- The three-point positioning algorithm based on acoustic emission sensors can realize the real-time and accurate positioning of the tank bottom detection robot, which overcomes the problem that the traditional positioning methods are limited in the confined space.
- The experiment shows that the average RMSE of RPF is 9.49% lower than that of IAPF (state-of-the-art). The visualization results show that RPF effectively reduces redundant path points compared with IAPF.

The rest of this article is structured as follows: Section 2 describes the proposed method. The experiment results and discussions are presented in Section 3. Finally, Section 4 is the conclusion.

## 2. Proposed Method

To achieve effective path planning and accurate positioning of the detection robot, first, the spiral preset path is designed according to the shape of the tank bottom. Then, the flow and parameter setting of the rotating potential field method are introduced. Finally, the principle of the positioning algorithm is described.

### 2.1. Rotating Potential Field Method Based on the Spiral Path

This work designs a spiral path for detecting the bottom of vertical cylindrical oil storage tanks in order to add the detecting area. Inspired by the motion mode of celestial bodies, we improve the traditional artificial potential field method through adding a rotation potential field (RPF) near the obstacles. The proposed method increases the ability of the artificial potential field method to jump out of local extreme points.

**Figure 1:**
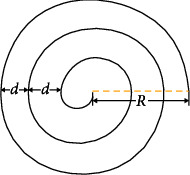
Schematic diagram of the robot spiral path.

First, the robot motion path is set to the spiral equation to make the detection path cover the bottom of the circular oil tank as much as possible. The spiral path is shown in Figure 1, and points on the path are expressed by *p*_*i*_(*x*_*i*_, *y*_*i*_). Points in the spiral path are by

(1) $$\begin{array}{c} P=\left\{p_{i}\right\},i=1,2,3,\ldots ,n_{\max}\text{,} \end{array}$$

where *n*_max_*is* the number of iterations required when the distance between the current position of the robot and the center of the circle is less than the threshold *d*_0_ or the preset maximum number of iterations is reached. The spiral path equation is written by

(2) $$\begin{array}{c} \left\{\begin{array}{c} x_{t}=\left(R-\frac{d\cdot t}{2\pi }\right)\cos\;\left(nt\right)\text{,}\\ y_{t}=\left(R-\frac{d\cdot t}{2\pi }\right)\sin\;\left(nt\right)\text{,} \end{array}\right. \end{array}$$

where *R* is the radius of oil tank bottom, *d* is the spiral coil pitch, *t* is the angular velocity, *n* is the number of iterations, and *n* ∈ [0, 2*πR*/*d*]. When detecting at the bottom, a point *p*_0_ on the tank wall is a starting point and the end point is *p*_*n*_max__.

**Figure 2:**
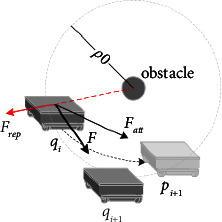
Force diagram of robot in the potential field.

**Figure 3:**
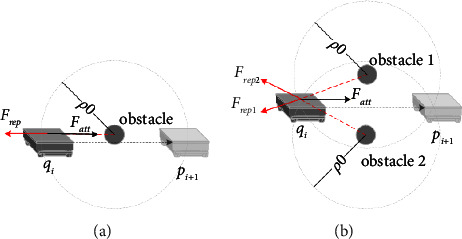
Examples of local extreme points in the potential field method (a) obstacle on the motion, (b) obstacles on both sides of the path.

On the spiral path, the traditional artificial potential field method is shown in Figure 2*Q*_*i*_ is considered as current location, and *p*_*i*+1_ is the target point. Point *q*_*i*_ is within the influence range of obstacles, that is to say, the distance between the point *q*_*i*_ and the obstacle is less than *ρ*_0_, where *ρ*_0_ is the influence radius of obstacles. The robot is affected by the gravitational *F*_att_ and repulsive *F*_rep_ at the same time, which makes the robot move along the direction of resultant force *F* and reach the point *q*_*i*+1_. When the resultant force is approach to zero, the path planning method based on the artificial potential field method is easy to fall into local extreme points.  Figure 3 shows an example of the case of falling into a local extreme point.

When it falls into the local extreme point on the spiral path, the potential field method is changing with the number of iterations. And the next target point is changed to a critical position. Although the path planning can jump out of the local extreme point, the robot still deviates from the spiral preset path, resulting in the failure of path planning.

**Figure 4:**
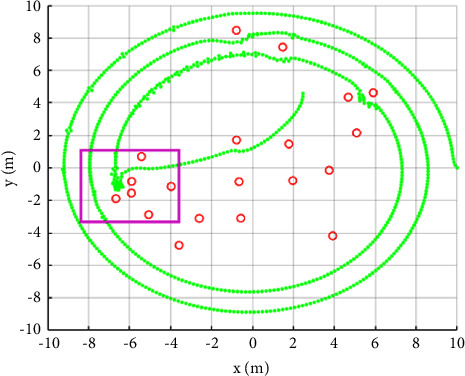
Schematic diagram of route planning failure caused by the artificial potential field method falling into local extreme points.

Figure 4 is a simulation experiment of the failure of path planning. If the radius of the circular bottom of the oil tank is 10, the starting point of the path planning is (10, 0) and the ending point is (0, 0). The robot moves counterclockwise according to the spiral path. The green points in the Figure 4 are the historical trajectory of the robot and the red points are obstacles. When the robot moves to the area marked by the purple box, it falls into local extreme points. Next, the results of multiple iterations are almost unchanged. After the next path point changes to a critical position, the robot jumps out of the local extreme point due to large gravity. But the path planning is a failure because the next path point deviates from the original preset route.

To solve this problem, the traditional artificial potential field method is improved in this article. Inspired by the motion mode of celestial bodies, we propose a rotating potential field method based on the spiral path through adding a rotating potential field near the obstacles.

The artificial potential field method is easy to fall into local extreme points because the resultant force is 0, which causes the robot to stop moving. The robot moves from the tank wall to the center of the circular tank bottom according to the spiral path. Similar to the movement of celestial bodies, the center of the circle is regarded as the central star, and the robot is taken as the planet moving around the central star. All obstacles are treated as stars; then, the whole system is a multistar system. If the robot moves along the spiral path, it can be regarded as the existence of orbit attenuation in the planetary orbit. Unlike the multistar system in astronomy, the stars in this system always remain stationary, and the two do not rotate around each other. The influence range of the obstacle is regarded as the gravitational range of the star, and the gravitational force is not considered outside the influence range.

Under this assumption, when the planet enters the gravitational range of another star *H* around the central star, the planet rotates around *H*. The gravity generated by the central star is greater. After turning to a certain degree, the planet leaves the gravitational range of *H* and re-enter the orbit around the central star, that is, the robot may bypass the obstacles.

**Figure 5:**
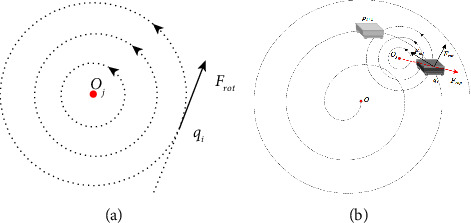
Schematic diagram of the rotating potential field (a) rotating potential field force. (b) Schematic diagram of force applied to the robot when the rotating potential field is increased.

**Figure 6:**
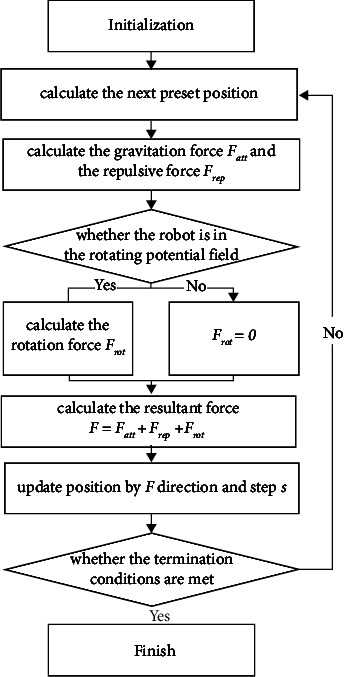
The flowchart of the rotating potential field method based on the spiral path.

In order to simulate this motion around the star, a rotating potential field is added around the obstacle and it is illustrated in Figure 5. When the current position point *q*_*i*_ is in the rotation potential field formed by the obstacle *O*_*j*_, the tangent direction of the circle with *O*_*j*_ as the center and passing through the *q*_*i*_ point at *q*_*i*_ is the gravitational direction. Since the rotation direction in the rotation potential field is counterclockwise, the tangent direction is also consistent with the rotation direction. Considering that the smaller the distance between *q*_*i*_ and *O*_*j*_, the robot may avoid the obstacle *O*_*j*_ as soon as possible and the greater the rotation potential field force. That is to say, the force of the rotating potential field should be inversely proportional to *ρ*(*q*_*i*_, *O*_*j*_). The gravitational force in the rotating potential field can be defined as

(3) $$\begin{array}{c} F_{rot}=\frac{K_{rot}\rho_{0}}{\rho \left(q_{i},O_{j}\right)}\text{,} \end{array}$$

where *K*_rot_ represents the gain coefficient of gravity in a rotating potential field and set *K*_rot_ = *K*_att_ in the experiment. The flowchart of the rotating potential field method based on the spiral path is shown in Figure 6.

### 2.2. Acoustic Emission Localization Algorithm

The tank bottom detection robot can sense the environment and its own state through sensors and further realize the target oriented autonomous movement in the environment with obstacles. In this work, we select acoustic emission sensors as the excitation source. Three base stations with known positions are set. The distances between the current position of inspection robot and the three base stations are measured through acoustic emission sensors. And then the three-point positioning algorithm is applied to realize the real-time calculation of the robot position.

**Figure 7:**
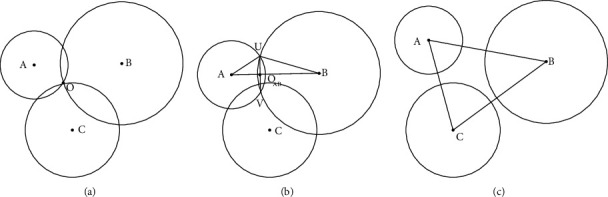
Schematic diagram of the three-point positioning algorithm (a) three-point positioning under ideal conditions; (b) three-point positioning where three circles all intersect; (c) three-point positioning where three circles do not coincide.

The three-point positioning algorithm [40] may adopt the position coordinate information of three known points to calculate the current position information. Here, the three points are not collinear.  Figure 7(a) represents the three-point positioning in an ideal situation. *O* is the current position of the acoustic emission sensor, and *A*, *B*, and *C* are three base stations. When the acoustic emission sensor is no error, the three circles with the radius of *OA*, *OB*, and *OC* have a unique intersection *O*. When the acoustic emission sensor exists error in the actual system, there are two cases, as shown in Figures 7(b) and 7(c). That is to say, the three circles may intersect in one area or the three circles may be separated.

For a group of base station locations *L* *=* {(*x*_1_, *y*_1_), (*x*_2_, *y*_2_), (*x*_3_, *y*_3_)} and the corresponding distances measured by acoustic emission sensor *D* = {*d*_1_, *d*_2_, *d*_3_}, the current position of the robot is (*x, y*). We can obtain the equation

(4) $$\begin{array}{c} \left\{\begin{array}{c} {\left(x_{1}-x\right)}^{2}+{\left(y_{1}-y\right)}^{2}=d_{1}^{2}\text{,}\\ {\left(x_{2}-x\right)}^{2}+{\left(y_{2}-y\right)}^{2}=d_{2}^{2}\text{,}\\ {\left(x_{3}-x\right)}^{2}+{\left(y_{3}-y\right)}^{2}=d_{3}^{2}\text{.} \end{array}\right. \end{array}$$

Equation (4) is simplified as follows:

(5) $$\begin{array}{c} \left\{\begin{array}{c} x^{2}+y^{2}-2x_{1}x-2y_{1}y=d_{1}^{2}-x_{1}^{2}-y_{1}^{2}\text{,}\\ x^{2}+y^{2}-2x_{2}x-2y_{2}y=d_{2}^{2}-x_{2}^{2}-y_{2}^{2}\text{,}\\ x^{2}+y^{2}-2x_{3}x-2y_{3}y=d_{3}^{2}-x_{3}^{2}-y_{3}^{2}\text{.} \end{array}\right. \end{array}$$

Subtract the third equation from the first two equations in equation (5) and it is written by

(6) $$\begin{array}{c} \left\{\begin{array}{c} \left(x_{3}-x_{1}\right)x+\left(y_{3}-y_{1}\right)y=\frac{\left(d_{1}^{2}-d_{3}^{2}+x_{3}^{2}+y_{3}^{2}-x_{1}^{2}-y_{1}^{2}\right)}{2}\text{,}\\ \left(x_{3}-x_{2}\right)x+\left(y_{3}-y_{2}\right)y=\frac{\left(d_{2}^{2}-d_{3}^{2}+x_{3}^{2}+y_{3}^{2}-x_{2}^{2}-y_{2}^{2}\right)}{2}\text{.} \end{array}\right. \end{array}$$

Let

(7) $$\begin{array}{c} X=\left[\begin{array}{cc} x_{3}-x_{1} & y_{3}-y_{1}\\ x_{3}-x_{2} & y_{3}-y_{2} \end{array}\right]\text{,}\\ \beta =\left[\begin{array}{c} x\\ y \end{array}\right]\text{,}\\ Y=\left[\begin{array}{c} \frac{\left(d_{1}^{2}-d_{3}^{2}+x_{3}^{2}+y_{3}^{2}-x_{1}^{2}-y_{1}^{2}\right)}{2}\\ \frac{\left(d_{2}^{2}-d_{3}^{2}+x_{3}^{2}+y_{3}^{2}-x_{2}^{2}-y_{2}^{2}\right)}{2} \end{array}\right]\text{.} \end{array}$$

Then, we can obtain the equation

(8) $$\begin{array}{c} X\beta =Y\text{.} \end{array}$$

So far, we may easily solve equation (8) by the least square method.

(9) $$\begin{array}{c} \beta^{\prime }={\left(X^{T}X\right)}^{-1}X^{T}Y\text{,} \end{array}$$

where *β*′ is the approximate value of *β*.

## 3. Experimental Test Results and Discussion

This section includes three parts. First, the proposed RPF method is compared quantitatively and qualitatively with the existing methods under different obstacle distributions. Next, the impact of different parameters in the RPF on the planning results is discussed and analyzed. Finally, the effectiveness of the three-point positioning algorithm is verified by the positioning experiment.

### 3.1. Path Planning Simulation Experiments

To verify the effectiveness of the path planning algorithm proposed in this article, the robot path planning simulation experiment is carried out. The CPU model used in the experiment is Intel (R) core (TM) i7-9750h CPU @ 2.60 GHz, and the simulation software platform version is MATLAB 2016.

In the experiment, we model the tank bottom as a circular surface with a radius of 10. The center of the circle is the origin of the coordinate axis. Green points are used to represent the robot's motion path. The motion start point is (10, 0) and the end point is (0, 0). The robot moves counterclockwise. The termination condition is that the number of iterations is reached or the distance from the end point is less than the threshold *d*_0_.

We randomly generate *m* obstacles to verify the path planning ability of the algorithm under different obstacle distributions. The *m* obstacles are shown as *O*_*j*_(*x*_*j*_, *y*_*j*_), *j* = 1, 2, 3, …, *m*, where *x*_*j*_ ∈ (−10, 10), *y*_*j*_ ∈ (−10, 10), and *x*_*j*_^2^+*y*_*j*_^2^ > *d*_0_^2^.

The common parameter settings of different algorithms are the same, where *K*_att_ = 200, *K*_rep_ = 200, the step size of each iteration move *s* is set as 0.2, the pitch of spiral path *d*=0.4*π*, angular velocity *t* *=* 0.02, and the distance in termination condition *d*_0_ is 0.2.

In order to quantitatively compare the effectiveness of path planning of different algorithms, we define three quantitative evaluation indexes, namely, precision (*P*), recall (*R*), and root mean square error (RMSE). Among them, *P* and *R* indexes are originated to machine learning indicators. For the path planning point *q*_*i*_ and its corresponding preset path point *p*_*i*_, it is regarded as the correct point at *ρ*(*q*_*i*_, *p*_*i*_) ≤ *T*_0_; otherwise, it is the wrong point. The three indicators are designed as follows:

Precision (*P*) [41] is estimated by

(10) $$\begin{array}{c} P=\frac{TP}{TP+NP}\text{,} \end{array}$$

where TP indicates the total number of correct points in path planning, NP is the total number of error points, and *P* is the proportion of correct points in all path points in the path planning.

Recall (*R*) [41] is estimated by

(11) $$\begin{array}{c} R=\frac{TP}{TP+FN}\text{,} \end{array}$$

where FN is the total number of planned errors on the preset path, and *R* is the proportion of correct points in all the preset path points in the path planning.

RMSE [42] is computed by

(12) $$\begin{array}{c} RMSE=\sqrt{\frac{1}{n_{\max}}\overset{n_{\max}}{\underset{i=1}{\sum }}\rho {\left(q_{i},p_{i}\right)}^{2}}\text{.} \end{array}$$

Multiple groups of obstacles are randomly generated within the given range. The existing methods and the proposed method are compared on three groups of obstacles with different distribution. The quantitative analysis is listed in Table 1. Both *P* and *R* indicators are counted at *T*_0_ = 0.2. The optimal indicators of each group are written in bold in Table 1. We can see that VT, IAPF (state-of-the-art), and RPF are successful in path planning under the influence of three groups of the obstacles with different distribution. Compared with the other two methods, RPF achieves better performance. Under the obstacle distribution 1, the RMSE index of RPF is reduced by 23.43% (from 34.79 to 26.64) compared with that of VT. Meanwhile, the precision of RPF is 6.46% (from 71.07 to 77.53) higher than that of VT. Under the obstacle distribution 2, the RMSE index of RPF is reduced by 3.46% (from 16.19 to 15.63) compared with that of IAPF. Under the obstacle distribution 3, the RMSE index of RPF is reduced (from 14.17 to 13.08) compared with that of IAPF. The average RMSE of RPF is 9.49% (from 20.20 to 18.45) lower than that of IAPF. In addition, combined with the improved repulsion (IR) function, VT, IAPF, and RPF have increased in all three indicators. Compared with IAPF + IR, RPF + IR achieves 6.41% (from 12.17 to 11.39) improvement in terms of RMSE under the obstacle distribution 3. Furthermore, the average RMSE of RPF + IR is 3.51% (from 18.54 to 17.89) lower than that of IAPF + IR. These comparisons verify the effective of RPF. Moreover, the precision of RPF + IR is 8.51% (from 77.53 to 86.04) higher than that of RPF under the obstacle distribution 1. Therefore, combining with the IR function can further improve the performance of RPF. We also compared the path planning time of different methods. The average planning time of RPF + IR is 0.40 s, which is very close to the average planning time of IAPF + IR of 0.38 s.

**Figure 8:**
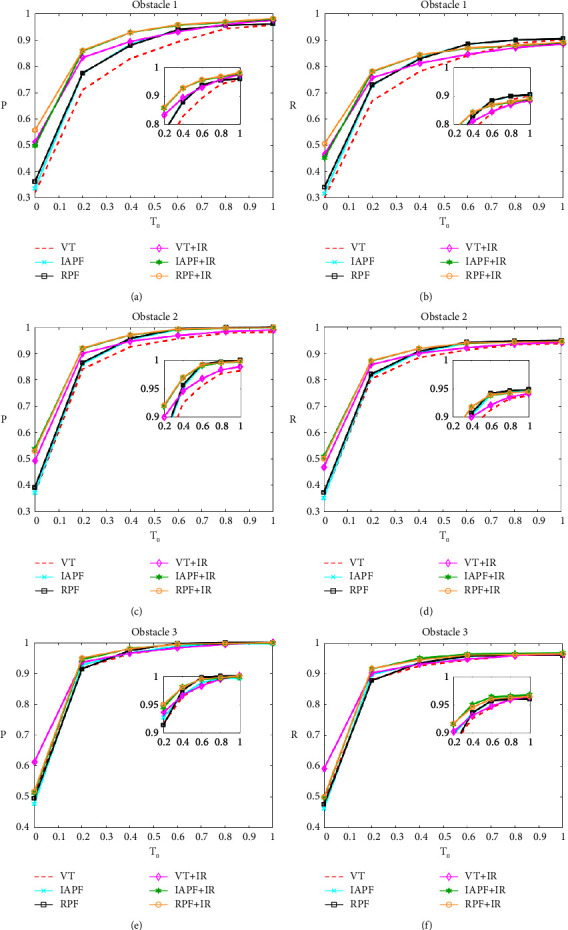
*P* and *R* curves of different algorithms at *T*_0_ = 0, 0.2, 0.4, 0.6, 0.8, and 1.0 under three obstacle distributions. (a) *P* curve of different algorithms under obstacle distribution 1, (b) *R* curve of different algorithms under obstacle distribution 1, (c) *P* curve of different algorithms under obstacle distribution 2, (d) *R* curve of different algorithms under obstacle distribution 2, (e) *P* curve of different algorithms under obstacle distribution 3, and (f) *R* curve of different algorithms under obstacle distribution 3.

A statistical analysis of *P* and *R* under different *T*_0_*s* is illustrated in Figure 8 in order to further compare the consistency between the path planning of different algorithms and the preset path. Among the three methods without the improved repulsion function, the rotating potential field method is better than the virtual target method. Combined with the IR function, when *T*_0_ = 0, the *P* and *R* indexes of all methods are improved. For example, under the distribution of obstacle 1, VT + IR improves the precision of 19.34% and the recall of 16.47%. This is because the IR function adds some gravitation, which makes the robot moving closer to the preset path. From the *P* and *R* curves, RPF + IR has better performance under different *T*_0_ under the three obstacle distributions.

The spiral path planning results illustrated represent intuitively the obstacle avoidance effect of different algorithms under three obstacle distributions.

**Figure 9:**
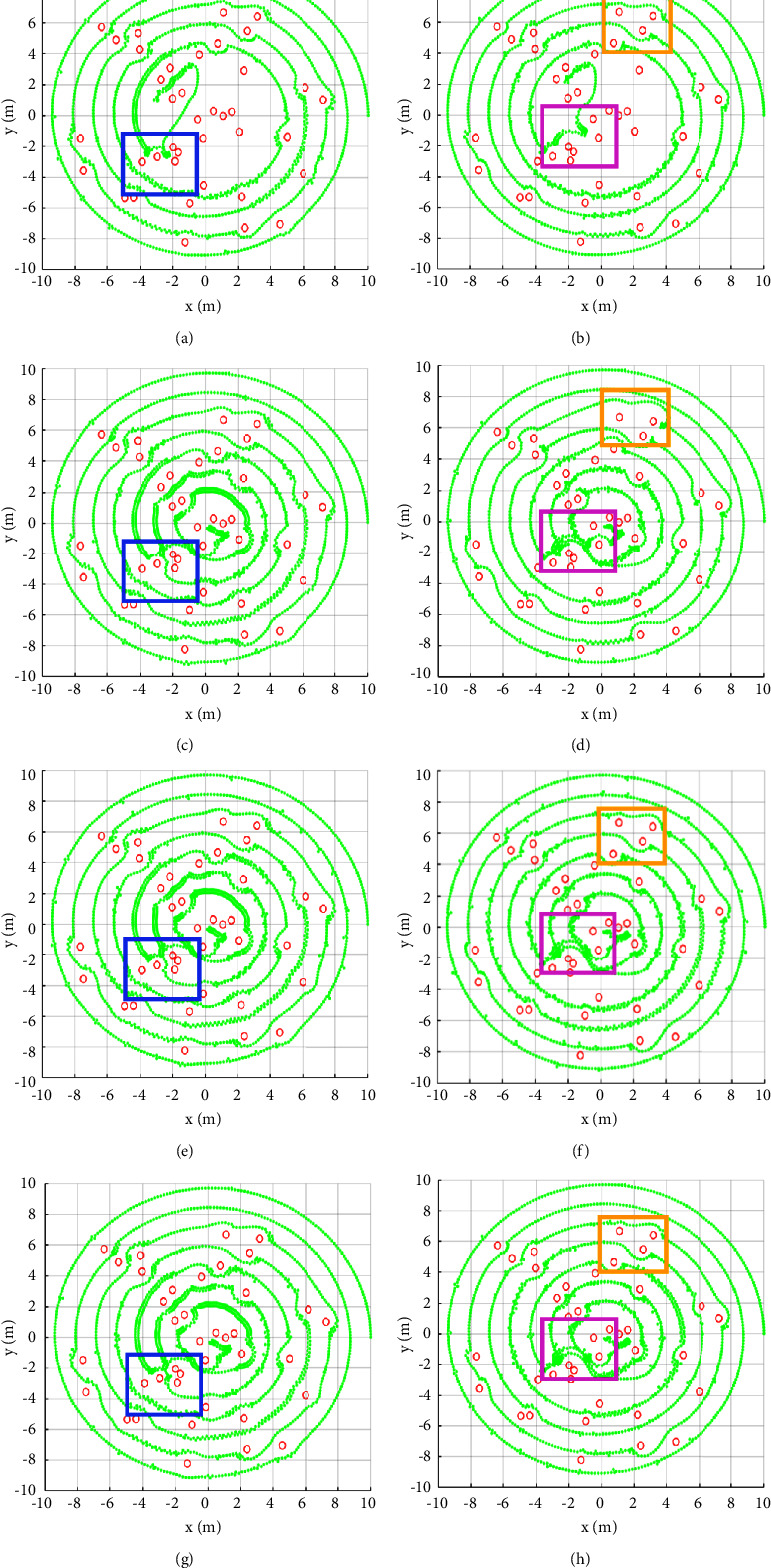
Path planning results of different algorithms under obstacle distribution 1. (a) APF, (b) APF + IR, (c) VT, (d) VT + IR, (e) IAPF, (f) IAPF + IR, (g) RPF, and (h) RPF + IR.

Figure 9 shows the results of different algorithms under obstacle distribution 1. In the blue rectangular box, it can be seen that APF failed to consider the local extreme points. While the path planning has been successfully realized in the VT, IAPF, and RPF. In the purple rectangular box, only APF + IR cannot successfully plan the path, but other methods combined with the improved repulsion may successfully plan the path.

From the yellow rectangular box, we can see that the overall planning of VT + IR is successful. But it falls into a local minimum point at trying to pass through the middle of two obstacles for many times before avoiding obstacles. The robot was not separated from the local extreme point until the gravity of a distant point on the preset path is large enough and a deviation between the obstacle avoidance path and the preset path is large enough. However, IAPF + IR and RPF + IR have effectively avoided the local minimum point. In addition, the planning results of IAPF + IR have redundant path points on both sides of the preset spiral path. And RPF + IR in our work does not have this problem.

**Figure 10:**
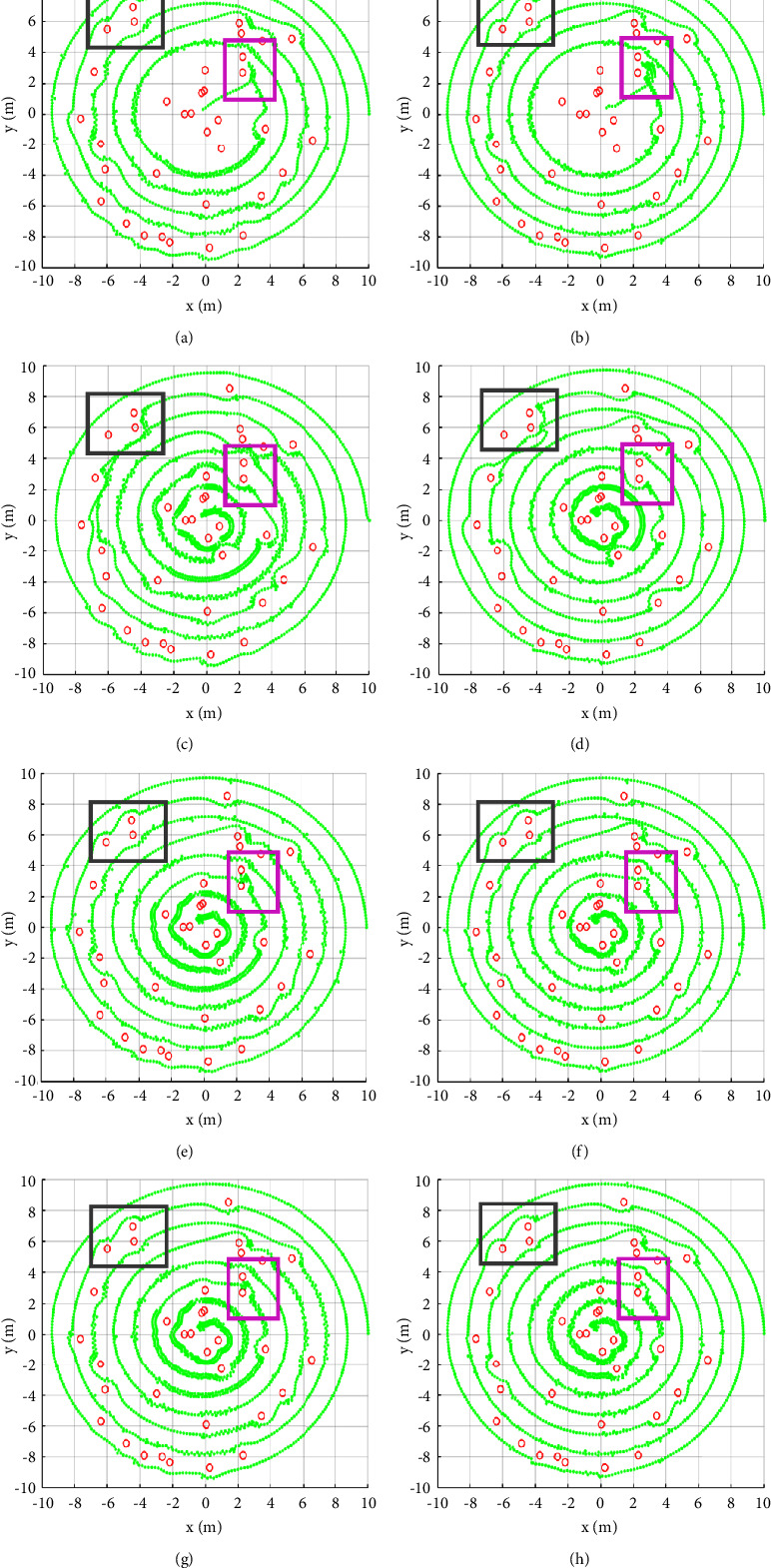
Path planning results of different algorithms under obstacle distribution 2. (a) APF, (b) APF + IR, (c) VT, (d) VT + IR, (e) IAPF, (f) IAPF + IR, (g) RPF, and (h) RPF + IR.

Figure 10 presents the results of path planning in obstacle distribution 2. By observing the gray rectangular box, we can see that the planning result of the VT is different from that of other methods. This is caused by the design of virtual points. Under the influence of the counterclockwise rotating potential field, RPF maintains an upward detour path. Combined with the IR function, different algorithms perform similarly in this local region.

In the purple rectangular region, both APF and APF + IR fail to plan and the other algorithms are successful. VT and VT + IR avoid two obstacles by detour, while IAPF, IAPF + IR, and RPF do not fall into the local minimum point under the action of the rotating potential field. They also cross successfully between the two obstacles, and these methods have better performance in quantitative analysis.

**Figure 11:**
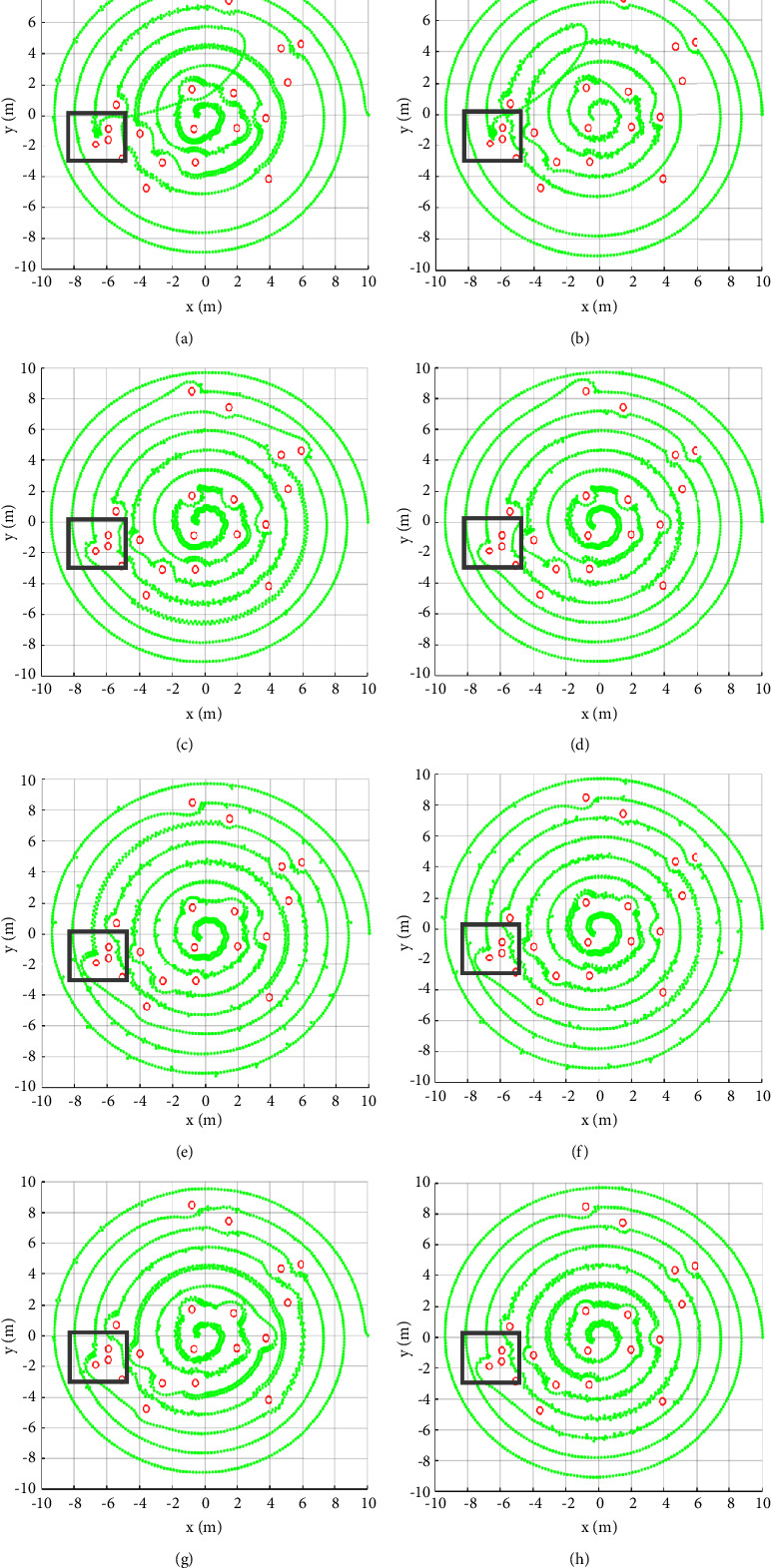
Path planning results of different algorithms under obstacle distribution 3. (a) APF, (b) APF + IR, (c) VT, (d) VT + IR, (e) IAPF, (f) IAPF + IR, (g) RPF, and (h) RPF + IR.

Figure 11 displays the path planning results of obstacle distribution 3. In the gray rectangular box, both APF and APF + IR fall into the local minimum point, resulting in the failure of path planning. Other methods may avoid obstacles successfully and complete the path planning. Moreover, the planning results of IAPF + IR have redundant path points on both sides of the preset spiral path. And RPF + IR does not have this problem.

**Figure 12:**
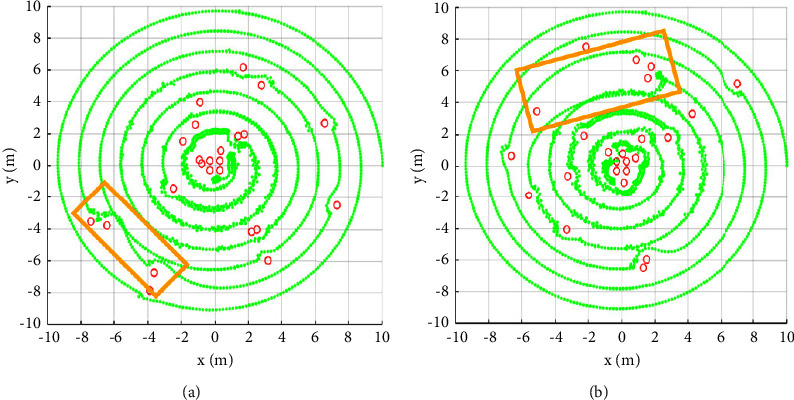
Poor results of RPF. (a) RPF results on obstacle 4 distribution (b) RPF results on obstacle 5 distribution.

We also analyzed the poor results of RPF in path planning. When the robot's current obstacle avoidance action has not ended and meets other obstacles, the obstacle avoidance path will deviate from the preset spiral path, as shown in Figure 12. When this situation occurs continuously, it will lead to more detection blind areas when the tank bottom detection robot performs the detection task as shown in the yellow rectangle in Figure 12. Obstacle avoidance is equivalent to increasing the distance from the current position to the target position, but the step length of the algorithm is fixed, and so more movements are required and the position of the target point is constantly updated. Therefore, when the obstacle avoidance action occurs repeatedly, the distance between the robot's current position and the target point is large, and the gravitation of the target point to the current position is strong. This makes the robot move rapidly towards the target point and cannot maintain the preset spiral path.

### 3.2. Influence of the Parameter Settings Experiment

In the proposed RPF method, its performance is affected by the setting parameters. We implement the path planning experiment under obstacle distribution 1 for different *K*_att_, *K*_rep_, *K*_rot,_ and *ρ*_0_. The 4 different *ρ*_0_*s* and 7 different ratios of *K*_att_ and *K*_rep_ are set in this experiment. The quantitative comparison results of different *ρ*_0_ are listed in Table 2. The difference between the best index and the worst index of *P* is 2.11% (from 88.07% to 85.96%). The difference between the best index and the worst index of *R* is 1.53% (from 79.85% to 78.32%). The difference between the best index and the worst index of RMSE is 0.0377 (from 0.2551 to 0.2928). These differences prove that different parameters have little influence on the success of RPF path planning.

### 3.3. Acoustic Emission Positioning Simulation Experiment

To verify the effectiveness of the three-point positioning algorithm, the simulation experiment of robot positioning is carried out in this section. In the experiment, three basis points are set up with the coordinates *A* (7.5, 0), *B* (−7.5, 0), and *C* (0, 7.5). A random point is given as the truth point (*x*_gt_, *y*_gt_), and the distance from the point to the three basis points is calculated. We add random disturbances to the three distance values to simulate the error in the actual system. If the distance from the truth point to the base station is *l*, the distance *l*_e_ after adding random disturbance is

(13) $$\begin{array}{c} l_{e}=\left(1+v\right)\cdot l\text{,} \end{array}$$

where *v* ∈ [−0.05, 0.05] stands for the random disturbance.

Since the acoustic emission sensor ranging results at the same location are different each time in the actual system, this experiment adds different random disturbances to the distance between the same truth point and the basis point many times. The average of the multiple positioning results determined the final position (*x*_*loc*_, *y*_*loc*_). In the experiment, the repetition times are set to 20 times. The Euclidean distance between the positioning result and the truth point is computed as the error *E* of the positioning algorithm.

(14) $$\begin{array}{c} E=\sqrt{{\left(x_{loc}-x_{gt}\right)}^{2}+{\left(y_{loc}-y_{gt}\right)}^{2}}\text{.} \end{array}$$

**Figure 13:**
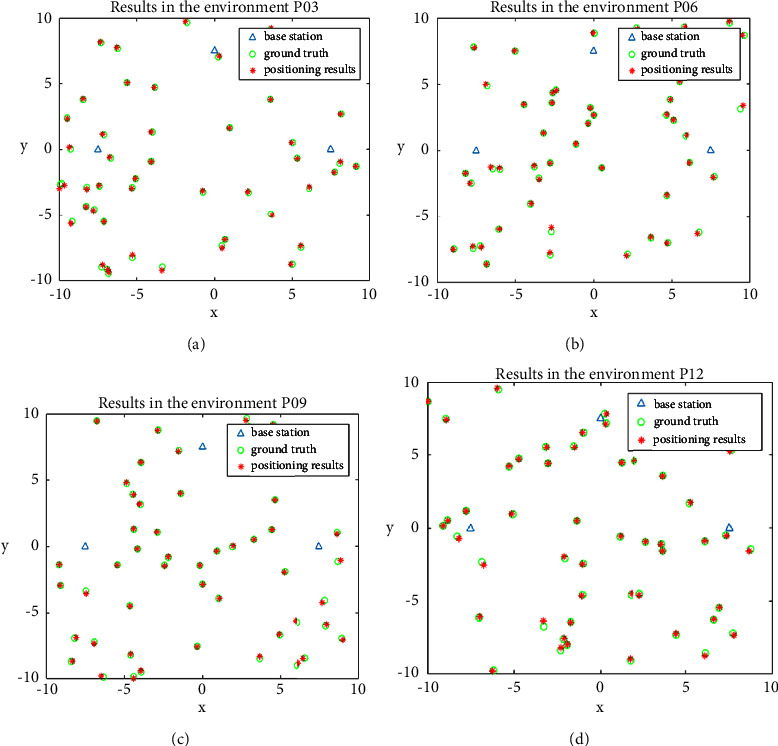
Three-point positioning algorithm visualization results in different environments with *|v|* ≤ 0.05 (a) results in the environment P03, (b) results in the environment P06, (c) results in the environment P09, and (d) results in the environment P12.

In this experiment, several truth points are randomly generated to verify the effectiveness of the algorithm. 12 test environments (P01 to P12) are constructed for the positioning accuracy test. Each environment contains 50 random points. We implemented the proposed algorithm 30 times for each test with different *v*. Best, mean, worst, standard deviation, and t-test represent the solution over independent 30 runs under each environment, respectively. The results are displayed in Table 3. The level 0.05 of significance is considered for the *t*-test. The results indicate that the three-point positioning algorithm has good accuracy. When |*v*| ≤ 0.05, the average error in all environments is only 0.0839. At the same time, the *p*-values are far smaller than the level 0.05 of significance. In addition, we also tested the calculation time of the algorithm. Each positioning takes only 6∼10 ms, which meets the needs of the actual project. In order to display intuitively the positioning effect, the positioning visualization results are illustrated in Figure 13. It can be seen that the positioning results of the algorithm are very close to the true value point.

**Figure 14:**
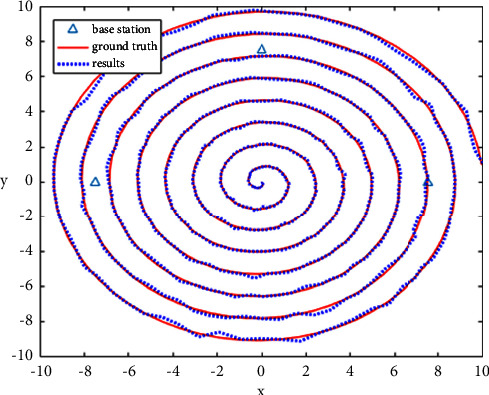
Schematic diagram of simulation results of the positioning algorithm on the spiral path.

In this article, the positioning simulation is also carried out according to the spiral path in the path planning. The starting point is still (10, 0), the spiral pitch *d*=0.4*π* and angular velocity *t* = 0.1, and a total of 500 points are generated. The three-point positioning algorithm is operated after adding random disturbance. The results are displayed in Figure 14. The trend of the algorithm results (blue points) is consistent with that of the preset spiral path (red curve). Under this kind of random disturbance, the average error of all point positioning results is only 0.0744. The 30 times with different random disturbance are run, and the average error is only 0.0748 ± 0.0032.

## 4. Conclusion

This work has investigated a rotating potential field method based on the spiral path for detecting the bottom of the tank. First, the preset spiral path was designed according to the shape of the tank bottom, and the rotating potential field was added on the basis of the artificial potential field method to achieve effective planning and obstacle avoidance. The average RMSE of RPF is 9.49% lower than that of IAPF (state-of-the-art), and the algorithm running time is not significantly reduced. After that, the three-point positioning algorithm was utilized to realize the calculation of the inspection robot position through measuring the acoustic emission range. The positioning error on the spiral path is only 0.0748 ± 0.0032.

The parameters in this article, such as *K*_att_, *K*_rot,_ and *K*_rep_, are selected based on experience, but different parameters do have certain impacts on the planning results, as discussed in Section 3.2. Therefore, better parameter selection can further improve the performance of RPF, and intelligent algorithms such as the genetic algorithm and the particle swarm optimization can be considered for parameter selection. In addition, we found in the simulation experiment that when the robot's current obstacle avoidance action has not ended and meets other obstacles, the obstacle avoidance path will deviate from the preset spiral path. When this situation occurs continuously, it will lead to more detection blind areas when the tank bottom detection robot performs the detection task. Therefore, how to solve the path deviation caused by the continuous obstacle avoidance action is an important issue to improve the algorithm's scene adaptability. Flexible step size design and direction constraints may solve this problem.

## Figures and Tables

**Table 1:** Comparison of path planning results of different algorithms under different obstacle distributions.

Methods	Obstacles 1 (*m* = 39, *ρ*_0_ = 1.5)			Obstacles 2 (*m* = 36, *ρ*_0_ = 1.0)			Obstacles 3 (*m* = 20, *ρ*_0_ = 0.8)		
	*P*↑ (%)	*R*↑ (%)	RMSE (×10^2^)	*P*↑ (%)	*R*↑ (%)	RMSE (×10^2^)	*P*↑ (%)	*R*↑ (%)	RMSE (×10^2^)
APF [24]	—	—	—	—	—	—	—	—	—
VT [33]	71.07	66.94	34.79	84.07	80.30	26.38	91.68	88.09	14.23
IAPF [34]	77.28	72.79	30.23	86.02	81.51	16.19	92.78	89.79	14.17
RPF (**Our**)	77.53	72.99	**26.64**	86.55	82.12	15.63	92.55	90.27	13.08
APF + IR [24]	—	—	—	—	—	—	—	—	—
VT + IR [33]	83.33	75.66	33.53	90.25	85.91	22.21	94.08	91.16	13.70
IAPF + IR [34]	85.78	77.96	29.48	91.94	87.08	13.97	94.54	91.64	12.17
RPF + IR (**Our**)	**86.04**	**78.16**	28.63	**92.11**	**87.20**	**13.65**	**95.06**	**91.76**	**11.39**

Bold indicates the best indicator.

**Table 2:** Comparison of RPF with different *ρ*_0_*s* and different ratios of *K*_att_ and *K*_rep_ under obstacle distribution 1 (bold indicates the best indicator and italics indicates the worst indicator).

*ρ*_0_	*K*_att_, *K*_rot_	*K*_rep_	*P* (%)	*R* (%)	RMSE
1.5	100	100	—	—	—
1.5	150	100	—	—	—
1.5	200	100	86.91	78.81	0.2858
1.5	225	100	86.39	78.44	0.2845
1.5	250	100	*85.96*	78.36	0.2881
1.5	275	100	86.22	*78.32*	0.2818
1.5	300	100	86.26	*78.32*	0.2739

2.0	100	100	—	—	—
2.0	150	100	—	—	—
2.0	200	100	86.97	79.25	0.2816
2.0	225	100	87.18	79.05	0.2638
2.0	250	100	87.19	79.17	0.2627
2.0	275	100	87.40	79.25	0.2588
2.0	300	100	87.12	79.17	0.2628

2.5	100	100	—	—	—
2.5	150	100	86.60	78.52	0.2878
2.5	200	100	**88.07**	**79.85**	0.2650
2.5	225	100	87.38	79.41	0.2626
2.5	250	100	87.24	79.49	0.2651
2.5	275	100	87.46	79.41	**0.2551**
2.5	300	100	87.35	79.45	0.2611

3.0	100	100	—	—	—
3.0	150	100	87.45	79.33	*0.2928*
3.0	200	100	87.78	79.77	0.2598
3.0	225	100	87.35	79.45	0.2605
3.0	250	100	87.25	79.57	0.2678
3.0	275	100	87.56	79.57	0.2562
3.0	300	100	87.31	79.41	0.2561

**Table 3:** Comparison of different *v* in 12 environments with 50 random points.

Environment	Statistics	|*v*| ≤ 0.01	|*v*| ≤ 0.02	|*v*| ≤ 0.03	|*v*| ≤ 0.04	|*v*| ≤ 0.05
P01	Best	0.0160	0.0319	0.0479	0.0650	0.0828
	Mean	0.0192	0.0388	0.0579	0.0734	0.0998
	Worst	0.0234	0.0465	0.0663	0.0831	0.1202
	Std. dev.	0.0019	0.0038	0.0051	0.0046	0.0100
	*t*-test	5.0225*e* − 31	4.9163*e* − 31	1.6554*e* − 32	8.4695*e* − 37	7.4859*e* − 31

P02	Best	0.0186	0.0329	0.0504	0.0754	0.0920
	Mean	0.0225	0.0432	0.0673	0.0900	0.1078
	Worst	0.0273	0.0580	0.0773	0.1062	0.1400
	Std. dev.	0.0021	0.0051	0.0068	0.0081	0.0099
	*t*-test	8.5476*e* − 32	7.9451*e* − 29	1.0632*e* − 30	4.0024*e* − 32	6.2938*e* − 32

P03	Best	0.0194	0.0342	0.0557	0.0737	0.0945
	Mean	0.0223	0.0440	0.0679	0.0898	0.1133
	Worst	0.0269	0.0505	0.0858	0.1068	0.1400
	Std. dev.	0.0018	0.0038	0.0070	0.0080	0.0125
	*t*-test	3.1414*e* − 33	9.5255*e* − 33	1.7882*e* − 30	2.6345*e* − 32	1.1984*e* − 29

P04	Best	0.0182	0.0354	0.0529	0.0646	0.0873
	Mean	0.0214	0.0433	0.0640	0.0864	0.1075
	Worst	0.0256	0.0492	0.0778	0.1026	0.1266
	Std. dev.	0.0017	0.0040	0.0059	0.0081	0.0106
	*t*-test	2.0551*e* − 31	1.0196*e* − 31	7.2237*e* − 32	1.0836*e* − 31	4.7596*e* − 31

P05	Best	0.0155	0.0351	0.0526	0.0756	0.0944
	Mean	0.0226	0.0432	0.0673	0.0885	0.1099
	Worst	0.0276	0.0492	0.0783	0.1021	0.1299
	Std. dev.	0.0026	0.0034	0.0068	0.0073	0.0084
	*t*-test	3.5962*e* − 29	1.0590*e* − 33	1.0840*e* − 30	2.9482*e* − 33	3.4386*e* − 34

P06	Best	0.0156	0.0307	0.0496	0.0590	0.0694
	Mean	0.0188	0.0386	0.0569	0.0740	0.0936
	Worst	0.0218	0.0479	0.0678	0.0905	0.1135
	Std. dev.	0.0016	0.0041	0.0051	0.0075	0.0105
	*t*-test	1.6495*e* − 32	5.2952*e* − 30	4.1761*e* − 32	1.0137*e* − 30	1.9780*e* − 29

P07	Best	0.0179	0.0323	0.0545	0.0709	0.0826
	Mean	0.0211	0.0421	0.0621	0.0842	0.1043
	Worst	0.0249	0.0524	0.0723	0.0962	0.1249
	Std. dev.	0.0019	0.0047	0.0046	0.0070	0.0088
	*t*-test	2.1113*e* − 32	2.1941*e* − 29	1.6340*e* − 34	4.1413*e* − 33	6.9580*e* − 33

P08	Best	0.0169	0.0339	0.0497	0.0644	0.0817
	Mean	0.0204	0.0397	0.0602	0.0790	0.0985
	Worst	0.0253	0.0498	0.0724	0.1019	0.1183
	Std. dev.	0.0021	0.0041	0.0060	0.0076	0.0079
	*t*-test	3.3745*e* − 30	1.4478*e* − 30	7.4149*e* − 31	2.2090*e* − 31	1.6295*e* − 33

P09	Best	0.0182	0.0345	0.0474	0.0727	0.0815
	Mean	0.0221	0.0430	0.0634	0.0848	0.1051
	Worst	0.0261	0.0475	0.0724	0.1114	0.1284
	Std. dev.	0.0018	0.0033	0.0059	0.0091	0.0113
	*t*-test	1.5502*e* − 33	2.6732*e* − 34	8.1635*e* − 32	5.5838*e* − 30	6.6782*e* − 30

P10	Best	0.0183	0.0363	0.0530	0.0714	0.0831
	Mean	0.0227	0.0454	0.0696	0.0907	0.1118
	Worst	0.0280	0.0537	0.0865	0.1057	0.1440
	Std. dev.	0.0022	0.0044	0.0081	0.0090	0.0133
	*t*-test	5.0725*e* − 31	3.0192*e* − 31	6.9498*e* − 29	6.3595*e* − 31	1.1045*e* − 28

P11	Best	0.0155	0.0328	0.0489	0.0587	0.0799
	Mean	0.0183	0.0379	0.0568	0.0737	0.0951
	Worst	0.0234	0.0456	0.0666	0.0912	0.1170
	Std. dev.	0.0018	0.0026	0.0053	0.0074	0.0091
	*t*-test	2.4976*e* − 31	1.2408*e* − 35	1.1776*e* − 31	9.2664*e* − 31	1.9567*e* − 31

P12	Best	0.0142	0.0298	0.0479	0.0677	0.0778
	Mean	0.0191	0.0394	0.0592	0.0784	0.0971
	Worst	0.0240	0.0439	0.0806	0.0989	0.1102
	Std. dev.	0.0024	0.0038	0.0073	0.0069	0.0079
	*t*-test	3.8579*e* − 28	2.4850*e* − 31	3.6348*e* − 28	1.9055*e* − 32	1.9444*e* − 33

## Data Availability

The datasets generated and/or analyzed during the current study are available from the corresponding author on reasonable request.
